# 3D Myocardial Scar Prediction Model Derived from Multimodality Analysis of Electromechanical Mapping and Magnetic Resonance Imaging

**DOI:** 10.1007/s12265-019-09899-w

**Published:** 2019-07-23

**Authors:** Hans Thijs van den Broek, Steven Wenker, Rutger van de Leur, Pieter A. Doevendans, Steven A.J. Chamuleau, Frebus J. van Slochteren, René van Es

**Affiliations:** 1 Department of Cardiology, University Medical Center Utrecht, Utrecht, The Netherlands; 2 Netherlands Heart Institute, Utrecht, The Netherlands; 3 CMH, Utrecht, Netherlands

**Keywords:** Heart failure, Myocardial infarction, NOGA, MRI, Electromechanical mapping, Feature tracking, Late gadolinium–enhanced MRI

## Abstract

Many cardiac catheter interventions require accurate discrimination between healthy and infarcted myocardia. The gold standard for infarct imaging is late gadolinium–enhanced MRI (LGE-MRI), but during cardiac procedures electroanatomical or electromechanical mapping (EAM or EMM, respectively) is usually employed. We aimed to improve the ability of EMM to identify myocardial infarction by combining multiple EMM parameters in a statistical model. From a porcine infarction model, 3D electromechanical maps were 3D registered to LGE-MRI. A multivariable mixed-effects logistic regression model was fitted to predict the presence of infarct based on EMM parameters. Furthermore, we correlated feature-tracking strain parameters to EMM measures of local mechanical deformation. We registered 787 EMM points from 13 animals to the corresponding MRI locations. The mean registration error was 2.5 ± 1.16 mm. Our model showed a strong ability to predict the presence of infarction (*C*-statistic = 0.85). Strain parameters were only weakly correlated to EMM measures. The model is accurate in discriminating infarcted from healthy myocardium. Unipolar and bipolar voltages were the strongest predictors.

**Electronic supplementary material:**

The online version of this article (10.1007/s12265-019-09899-w) contains supplementary material, which is available to authorized users.

## Introduction

For many cardiac catheter interventions, accurate discrimination between healthy and infarcted myocardia is crucial [1]. For example, ablation of ventricular tachycardia may require ablation lesions to be placed in and around the myocardial infarction (MI) area to eliminate electrical signals conducted via viable cells contributing to the arrhythmia [2]. Furthermore, in trials evaluating cardiac regenerative therapy, therapeutics are often targeted specifically to the infarct border zone [3]. The current gold standard for infarct imaging is late gadolinium–enhanced magnetic resonance imaging (LGE-MRI). Pre-procedural LGE-MRI may inform operators about the location of the diseased areas, but reviewing MRI data pre-procedurally is not sufficient to ensure accurate identification of target tissue intra-procedurally [4]. Both interventional MRI [5] and image fusion techniques [6, 7] are active fields of research that offer the possibility of incorporating MR imaging during interventional procedures. However, MRI guidance for cardiac treatment is currently not widely available and, therefore, interventional cardiologists in clinical practice have to rely on other techniques to identify target tissues intra-procedurally.

Within electrophysiology, electroanatomical mapping (EAM) is the standard technique to identify the origin of arrhythmia and to distinguish healthy from scarred myocardium [8, 9]. This technique is performed using a mapping catheter that is positioned inside the left ventricle (LV) and that is able to measure local electrical characteristics of the myocardium [10]. Using three magnetic fields, the system is able to deduct the position of the catheter and register the measurements to a 3D location. Electromechanical mapping (EMM) is an extension of this technique that allows for measurements of local mechanical properties as well [11]. Using these measurements, a 3D electromechanical map of the LV can then be constructed.

Previous research evaluated the ability of (individual) EAM/EMM parameters to discriminate between areas of MI and healthy tissue, using LGE-MRI as the gold standard [12–15]. In practice, MI is not a dichotomous phenomenon. Interspersed between areas that are healthy and areas that are completely infarcted (transmurally infarcted) are often areas in which the infarction does not extend completely through the myocardial wall (non-transmural infarction). Research has shown that different individual EMM parameters identify different regions of the MI, and the threshold of infarct transmurality at which the parameters offer the best diagnostic accuracy differs between the varying parameters [13–15]. For example, one study found that unipolar voltage (UV) best discriminates at a threshold of 5% infarct transmurality, while bipolar voltage (BV) has the highest diagnostic accuracy at a threshold of 97.5% transmurality [15]. Because MI is heterogeneous of nature and individual EMM parameters enable the differentiation of distinct regions, we propose that a prediction model that incorporates multiple EMM parameters could improve the detection and differentiation of MI.

Strain analysis, MRI feature tracking (MRI-FT), allows quantification of myocardial deformation on MRI. While other advanced deformation imaging methods require additional, often time-consuming imaging sequences, FT is based on standard cine MR imaging sequences that are routinely acquired. The reduced mechanical activity coincides with myocardial scar on LGE-MRI [16] and is even a sensitive marker for sub-clinical myocardial dysfunction [17]. However, as of yet, there is no data comparing the resulting strain parameters with EMM-derived parameters of local mechanical activity (local linear shortening (LLS) and local activation time (LAT)).

In this retrospective study, we investigated the use of a logistic prediction model based on multiple EMM parameters to distinguish infarcted from healthy myocardium with the most accuracy, and we evaluated the predictive accuracy of this model in a porcine model of chronic MI. Furthermore, we compared the EMM-derived parameters of local mechanical activity with MRI-FT–derived parameters.

## Methods

### Animals

We re-analyzed 13 EMM and MRI datasets acquired in a porcine model of chronic MI. The experiments have been described in more detail previously [15, 18]. In short, MI was induced by 90-min occlusion of the left anterior descending coronary artery distal to the second diagonal branch.

### Data Acquisition

#### Electromechanical Mapping (EMM)

The EMM procedure was described in detail previously [15]. In short, the NOGA® XP system (Biosense Webster, Johnson & Johnson, Diamond Bar, USA) was used to create an EMM of the LV by using a conventional 7 French deflectable-tip mapping catheter (NogaStar, Biosense Webster). Under fluoroscopy guidance, the catheter was introduced into the LV after retrograde passage through the aortic valves. To ensure complete LV endocardium coverage, EMM parameters (UV, BV, LAT, and LLS) were recorded at 80 to 200 LV endocardial locations. Electrocardiograms were filtered at 30–400 Hz (bipolar) and 1–240 Hz (unipolar). EMM measurement points were accepted if they were triggered on the R-wave in combination with acceptable catheter stability, in accordance with the criteria for good electromechanical mapping [11].

#### MRI Acquisition

Detailed acquisition settings can be found in the appendix. CMR images were acquired using a 1.5-T Ingenia system and a 3-T Achieva XT system (Philips Healthcare, Best, The Netherlands). Imaging planes were selected according to standard cardiac views (four-chamber, two-chamber, and short-axis view). Fifteen minutes after injection of 3.0 mmol/kg gadolinium, an LGE inversion recovery sequence was acquired in the short-axis orientation.

### Data Processing

#### Image Segmentation

LV segmentations were created using Segment for Matlab (version 2.1 R5768) [19]. Automatic segmentation of the epicardial and endocardial contours was performed on the short-axis cine images in the end-diastolic phase and end-systolic phase. Segmentation quality was visually assessed using long-axis images and adjusted if necessary. Wall thickness was measured on the short-axis cine images. Wall thickening (WT) was calculated as the difference between wall thickness in the end-diastolic and end-systolic phases. Fractional wall thickening (WT%) was calculated relative to the end-diastolic phase wall thickness.

Automatic identification, segmentation, and quantification of LGE lesions were performed using the full-width at half maximum (FMWH) algorithm. Area-based infarct transmurality (TM), WT, and WT% values were calculated in 80 circumferential segments per slice. In all image processing steps, manual correction was performed if necessary.

#### Strain Analysis

Myocardial deformation analysis was performed on short-axis cine images using FT software (TomTec Arena, 2D Cardiac Performance Analysis MR, version 1.2, Unterschleissheim, Germany). Circumferential strain curves were exported into a custom Matlab script to automatically determine time-to-peak-strain (TTP_max_) and maximum strain (strain_max_) for 48 sectors per slice. Identification of the timing-of-onset (*T*_onset_) was performed using a modified algorithm for estimation of the onset time of shortening as described in the appendix [20]. To prevent erroneous strain values in akinetic sectors affected by MI due to poor tracking of the image features, sectors with mean strain values below a pre-determined − 7.5% threshold were excluded from the strain analysis. The sectors were included in the univariable linear analysis as akinetic sectors.

#### Image Registration

**Fig. 1:**
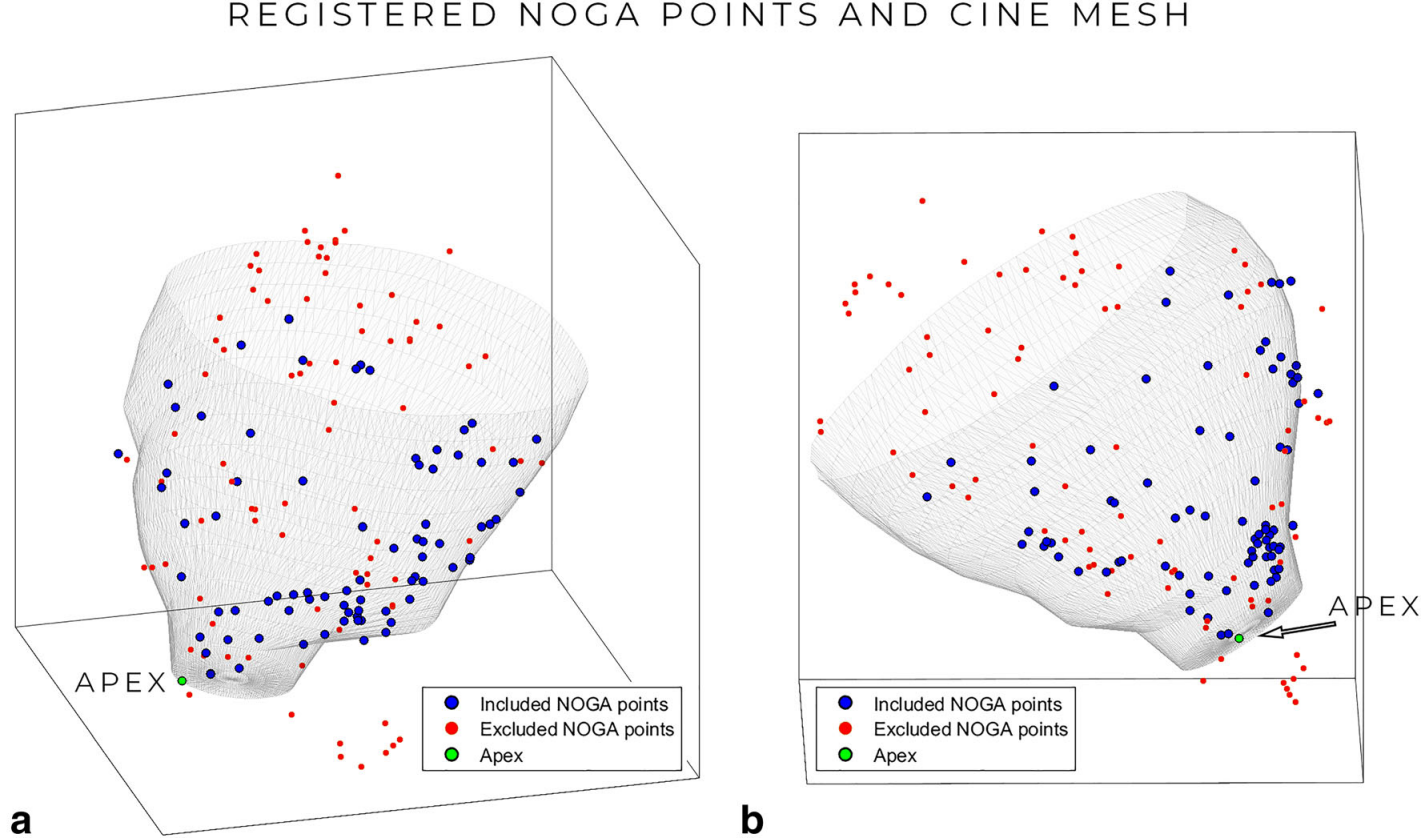
Projection of the NOGA-derived EMM-points on the endocardial surface mesh created from the MR images in LAO (**a**) and RAO (**b**) view. Red points are excluded based on their distance (> 5 mm) to the mesh

A 3D surface mesh was constructed from the end-diastolic LV cine MRI segmentation. The EMM points were registered to this mesh based on iterative closest point (ICP) algorithm [21]. The registration was manually optimized if necessary. Figure 1 shows an example of the registration of the LV endocardial mesh with EMM points. The algorithm and registration steps have previously been described in detail [22]. Registration error was expressed as the shortest distance of each EMM point to the closest point on the mesh surface. As a first step of the registration, both datasets where placed in patient coordinates, and the apex of both the EMM data and the 3D surface mesh was registered. Furthermore, during the ICP registration, the rotation parameter was constrained to 10°. EMM points that were located more basal than the most basal segmentation of the MR images (and therefore also outside of the mesh) were excluded.

After the EMM-MRI registration, MRI-derived (WT, WT%, TM) values were calculated for each EMM point as an inverse distance weighted average of all vertices of the surface mesh within 5 mm. EMM points without vertices within the 5-mm range were excluded for the model.

### Statistical Analysis

#### Scar Prediction Model

Infarct transmurality was dichotomized at a threshold of 50%. A multivariable prediction model for scar location on MRI with the EMM parameters as input was fitted using a logistic mixed-effects model with a random intercept per subject. All EMM parameters were scaled and centered and used as predictors in the model. Backward predictor selection was performed using Akaike’s Information Criterion (AIC), and 95% confidence intervals around the odds ratios were derived using 500 bootstrap samples for all models [23].

Predicted probabilities for scar were derived using the fixed effects and a mean random intercept since the random cluster effect will be unknown for new subjects. Performance of the prediction model (i.e., discrimination and calibration) was assessed using the *C*-statistic (equivalent to the area under the receiver operating curve) and calibration slope. The within-subject *C*-statistic was estimated as a mean of the *C*-statistic for each subject (i.e., each animal), weighted for the amount of EMM points per subject [24]. The within-subject calibration slope was derived from a logistic mixed-effects model with a random slope for the linear predictor and random intercept per subject [25]. Considering the small number of subjects, internal validation and correction for optimism in performance were performed using bootstrapping (*n* = 500) of individual data points (in contrast to bootstrapping complete clusters as would be preferable with a large number of subjects). A more comprehensive description of the internal validation approach can be found in supplementary information 2.

### Correlating EMM Parameters to Feature Tracking

A univariable linear mixed-effects model, with a random intercept per subject, was fitted for a one-to-one comparison of EMM parameters with MRI parameters. A fixed-effect *R*^2^ value is a statistical measure that represents the reduction in residual variance after adding parameters to a null model with only the random intercept. We approximated these values using a method described by Snijders and Bosker [26].

All statistical analyses were performed using *R* (version 3.5, R Foundation for Statistical Computing, Vienna, Austria) [27] and the *lme4* package [28]. Data are presented as mean ± SD or median with interquartile range (IQR) where appropriate. Point estimates are presented with a 95% confidence interval in square brackets. A *p* value of < 0.05 was considered statistically significant.

## Results

### Imaging

Chronic MI was present in all animals at the time of the EMM procedure as evidenced by a clear hyperintense area on LGE-MRI. In all animals, the infarctions were located apicoseptal and mid-apicoanterior. The MRI results are summarized in Table 1.

**Table 1:** Results of cine and late gadolinium enhancement magnetic resonance imaging of 15 animals

LV volumetry	
LV end-diastolic volume	110.7 ± 20.2 (ml)
LV end-systolic volume	59.5 ± 17.1 (ml)
LV ejection fraction	47.3 ± 9.7 (%)
Heart rate	54 ± 8 (bpm)
LV mass	118.2 ± 20.5 (g)
Infarct mass	16.8 ± 6.5 (g)
Infarct size	28.3 ± 12.3 (%)

All results are presented in mean ± standard deviation

After dichotomization, the mean endocardial surface area for MI was 18.5 ± 8.2 cm^2^ compared with 66.6 ± 10.2 cm^2^ for healthy tissue, thereby covering 21.2 ± 8.4% and 78.8 ± 8.4% of the total LV, respectively. The mean infarct volume was 17.2 ± 5.3 cm^3^.

An average of 49.1 ± 25.3 sectors were filtered in the strain analysis and marked as akinetic sectors, thereby covering 13.2 ± 6.9% of the total LV. In healthy tissue, the median circumferential strain_max_ was − 26.0% (IQR = − 34.9, − 17.6) and within myocardial scar the median value was − 15.7% (IQR = − 25.3, − 9.0).

### Image Registration

EMM points were homogeneously distributed over the LV endocardial surface. The total number of EMM points in the 13 datasets after filtering was 1459 (112 ± 41 per subject) and these were used for registration and projection. Of the registered points, 672 (46.1%) were not located within 5 mm of the mesh and were excluded from the model. The resulting registration error, after exclusion of these points, was 2.5 ± 1.2 mm. Ultimately, high-density maps with a total of 787 (61 ± 19 per subject, on average 5.0 ± 1.6 points per segment) points were matched to corresponding MR-derived values and were used for fitting the model.

### Linear Relationships Between EMM and LGE-MRI

We assessed the relationship between *T*_onset_, TTP_max_, and LAT time as well as between (fractional) WT, strain_max_, and LLS. Results from the univariable linear regression analysis are shown in the supplemental data**.** Both T_onset_ and TTP_max_ were significantly correlated with LAT (*p* = 0.05 and *p* = 0.02, respectively) and WT, fractional WT, and strain_max_ were all statistically significant predictors of LLS (*p* < 0.001 for all); although in all these correlations, the explained variance was small (*R*^2^ values ranged between 0.006 and 0.029).

### Prediction Model

**Fig. 2:**
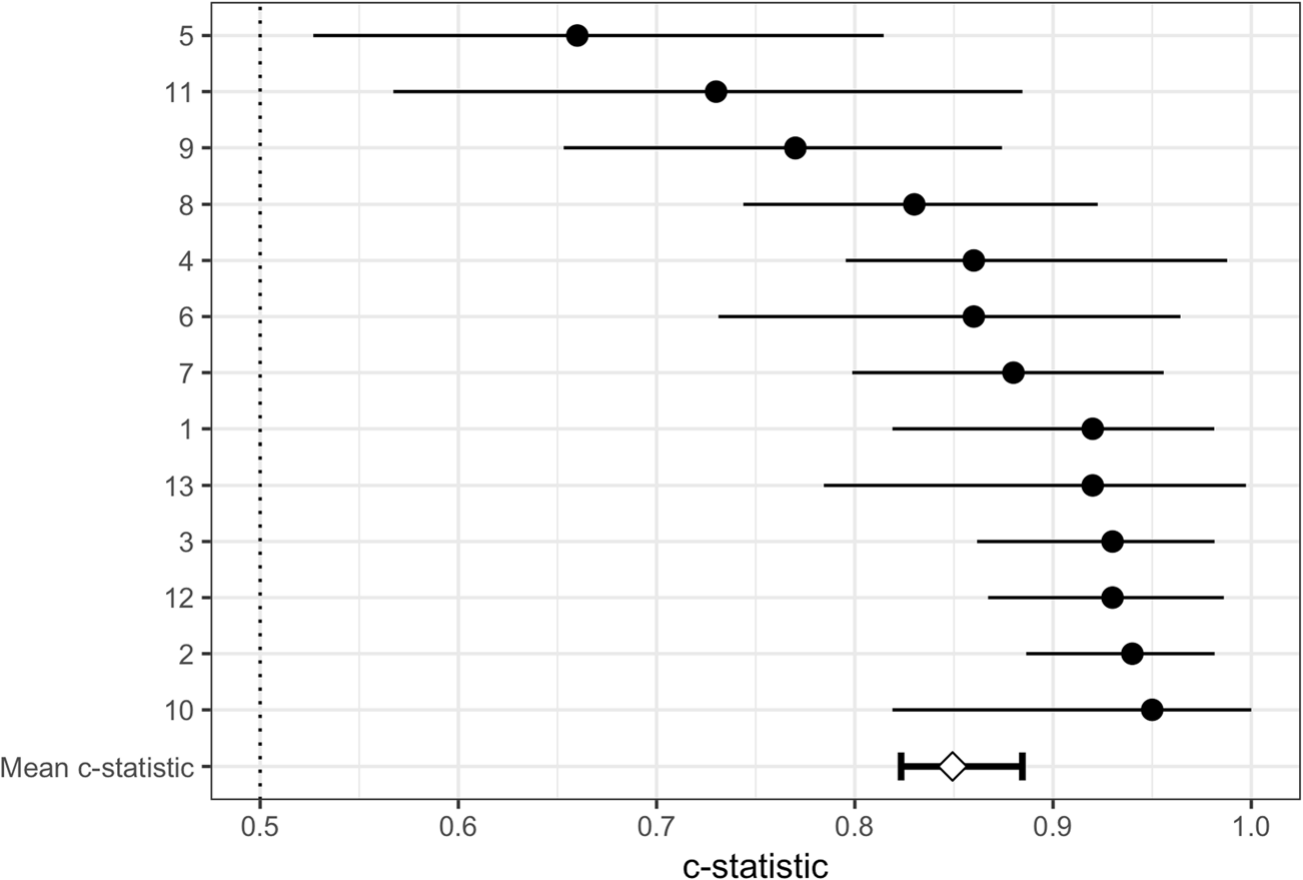
The within-subject model discrimination with 95% CI and the mean weighted C-statistic. Subject numbers are shown on the Y-axis

The odds ratios (OR) of the normalized EMM parameters are shown in Table 2. UV, BV, and LAT were statistically significant predictors for the presence of myocardial scar on LGE-MRI. In our dataset, UV was the strongest predictor for myocardial scar (OR = 0.14 [0.08–0.21]) followed by BV (OR = 0.36 [0.23–0.52]). The association with scar was less pronounced for LLS (OR = 0.76 [0.61–0.92]) and did not reach statistical significance for LAT (OR = 0.80 [0.61–1.06]). The combination of UV, BV, LLS, and LAT shows a strong predictive ability to discriminate between scar and no scar (*C*-statistic = 0.85 [0.82–0.89]). Internal validation of the prediction model showed a comparable optimism-corrected *C*-statistic value of 0.84 and good calibration (calibration slope = 1.01), as shown in Table 3. Within-subject results of the multivariable logistic mixed model (evaluated without the random intercept) can be appreciated from Fig. 2. The sensitivity and specificity of the combined EMM parameters to distinguish between scar and no scar were 72% and 85%.

**Table 2:** Odds ratio results of the multivariable logistic mixed model analysis for four EMM parameters

Parameter	Odds ratio
Unipolar voltage	0.14 [0.08–0.21]
Bipolar voltage	0.36 [0.23–0.52]
Local linear shortening	0.76 [0.61–0.92]
Local activation time	0.80 [0.61–1.06]

All results are presented in odds ratio with 95% confidence interval

**Table 3:** Internal validation results of the multivariable prediction model

Original *C*-statistic	0.85 [0.82–0.89]
Optimism-corrected *C*-statistic	0.84
Original slope	1.10 [1.00–1.21]
Optimism-corrected slope	1.01

Data is presented as the point estimate with 95% confidence interval

**Fig. 3:**
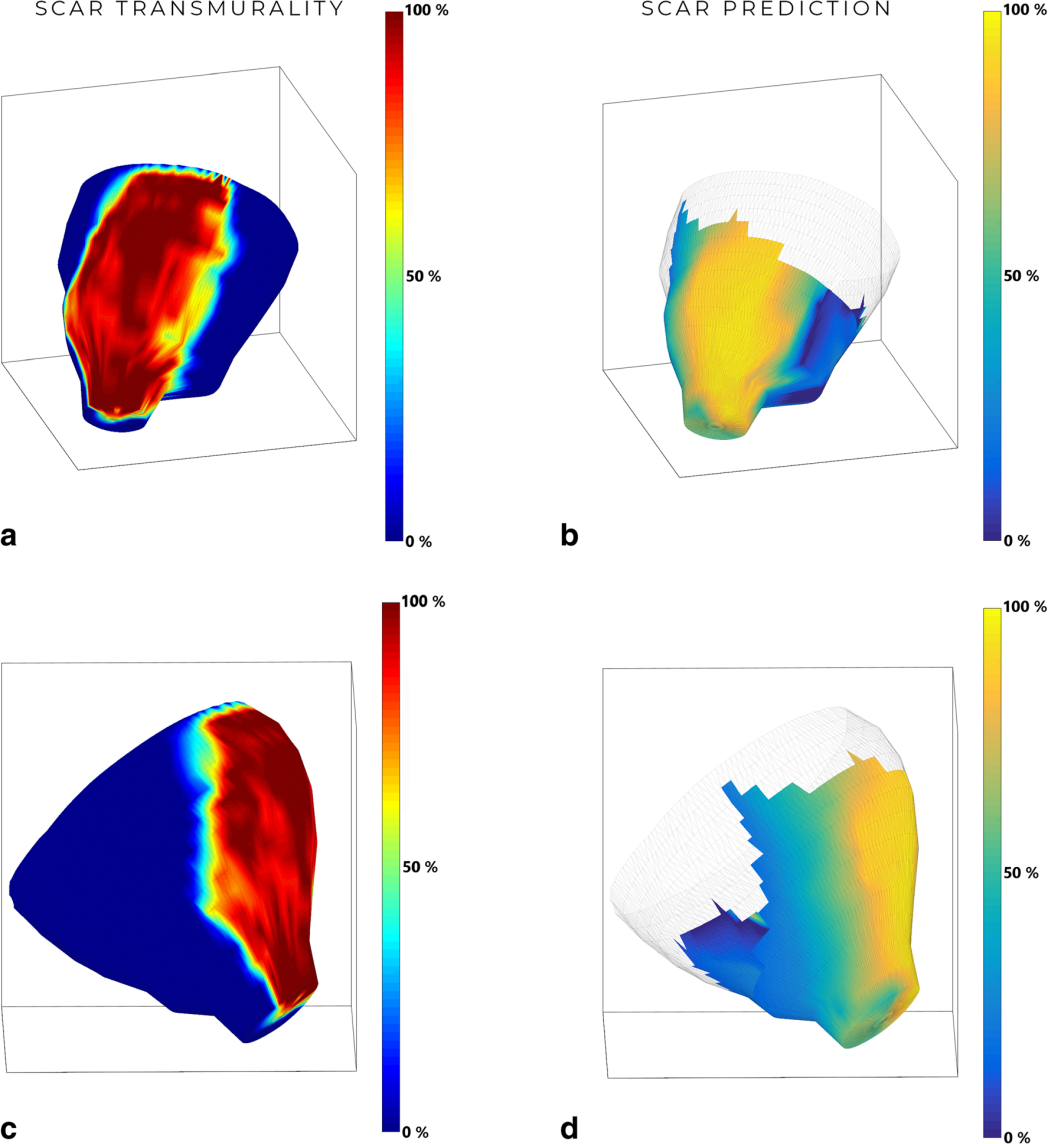
The LGE-MRI–derived scar transmurality versus the NOGA-predicted scar model for animal 2. **a**, **c** LGE-MRI–derived myocardial infarct transmurality projected on a cine surface mesh. The values of the scar transmurality are reflected in the color bar. **b**, **d** Predicted scar transmurality based on EMM-derived parameters projected on a cine surface mesh. The values of the predicted scar transmurality are reflected in the color bar

**Fig. 4:**
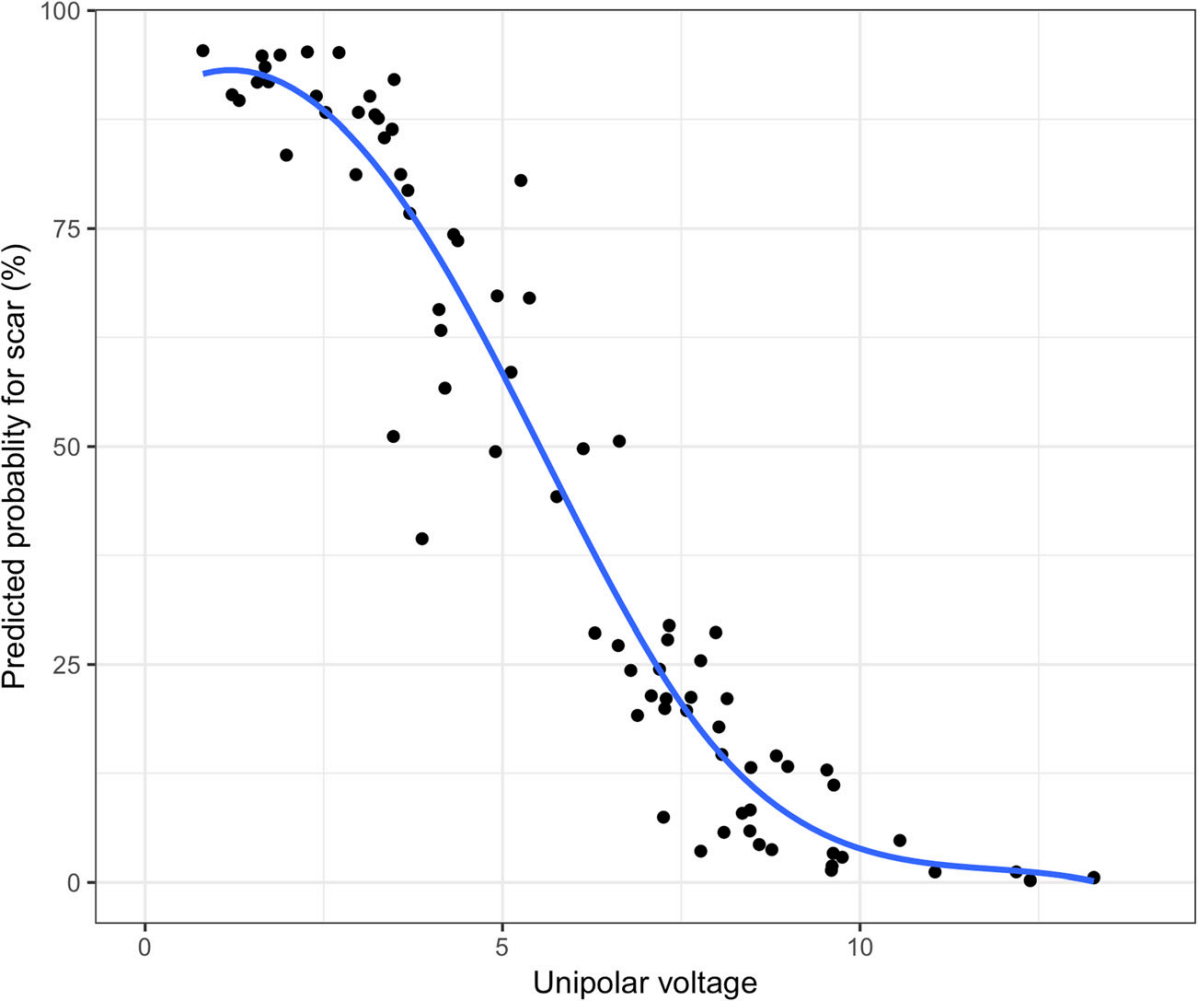
Predicted probability plot for myocardial scar determined by unipolar voltage for one of the animals (subject 2)

**Fig. 5:**
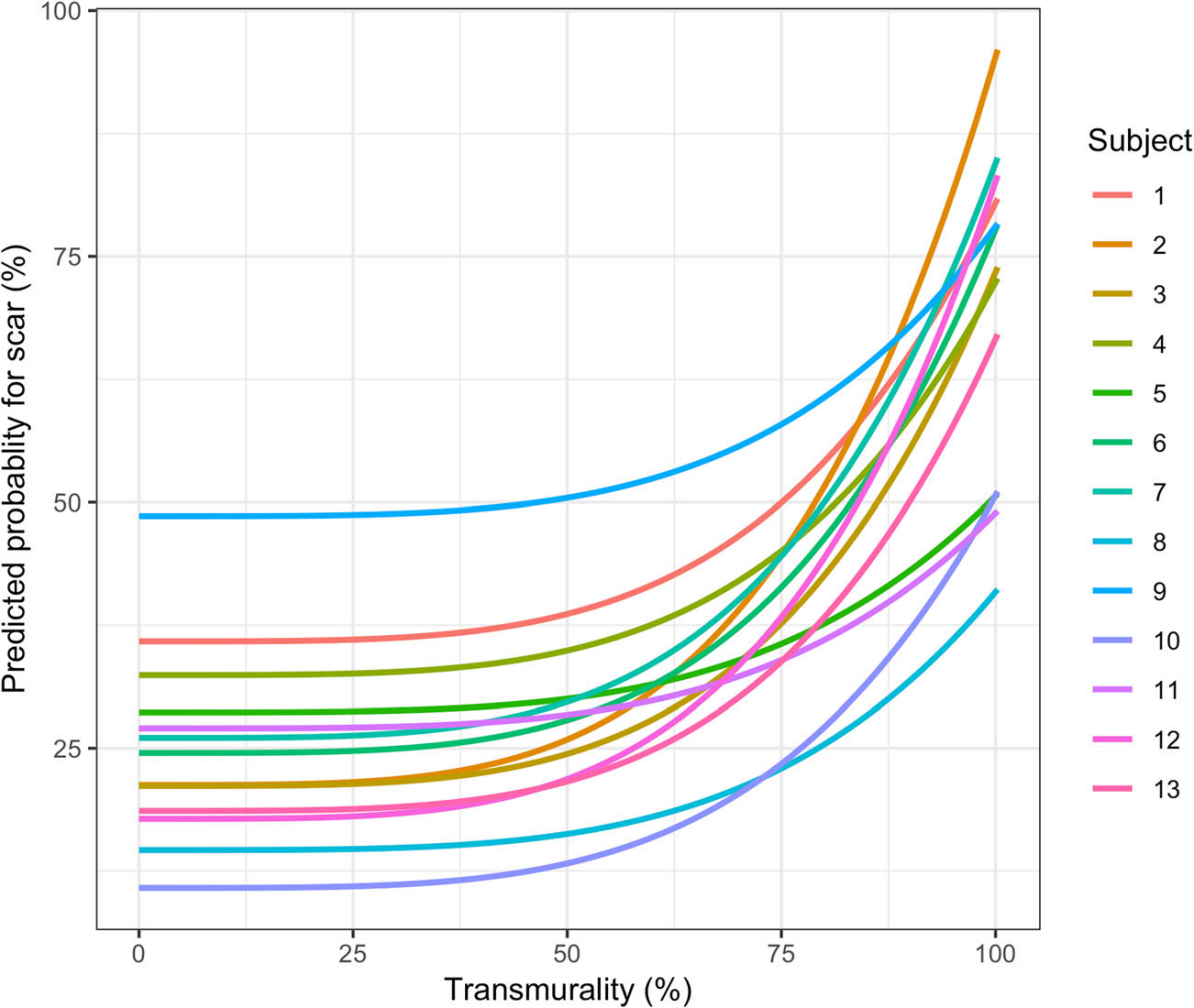
Relationship between the predicted probabilities of the model and the actual transmurality percentage

The relationship between the predictions made by our model and the presence of scar on MRI can visually be appreciated from Fig. 3, which shows both the MRI-derived values plotted on the mesh (a and c) and the prediction of our model (b and d) for one of the animals. The predicted probabilities of scar, for the same animal, are plotted in Fig. 4 as a function of unipolar voltage. Figure 5 shows the relationship between the predicted probability of the model and the actual transmurality.

## Discussion

In this study, 13 NOGA and MRI datasets of a porcine model of chronic MI were retrospectively analyzed. Parameters derived from electromechanical mapping (UV, BV, LAT, LLS) were registered in 3D and combined with MRI parameters in a multivariate mixed model for prediction of myocardial scar. The animal model used in this study provided large transmural myocardial infarct areas with only small non-transmurally infarcted areas. To prevent the effect introduced by the variation of samples measured in the small non-transmurally infarcted areas on the model, the outcome parameter was dichotomized to transmural infarction or healthy myocardium and logistic regression was fitted. Consequently, the resulting scar prediction model has the highest predictive ability to discriminate between infarcted and non-infarcted myocardia.

The accuracy of the electromechanical map depends on the number, quality, and distribution of the measurements acquired. A homogenous distribution of EM points importantly determines the result of the interpolation between measurement points and thus the accuracy of the 3D map. Because the NOGA system only acquires a single point per measurement, the mapping procedure requires time and patience to create complete and representative maps. A strength of our study is the high density of the acquired NOGA maps. In all animals, the electromechanical maps were more densely populated than the recommended minimum of 3 points per cardiac segment [11] allowing accurate comparison to MR images over a large number of sampled points. Even though electromechanical maps with an overall high number of points were used in this study, the shape of the EMM surface mesh is interpolated between the measured points and, therefore, never exactly represents the LV endocardial surface. Furthermore, because MR and EMM are not performed at the same time, hemodynamic and LV filling conditions may have changed between the two individual data acquisitions. Aforementioned factors inevitably result in a registration error.

To fit a statistical model that predicts transmurality from EMM parameters, it is important to match the EMM parameters to the transmurality values of the same location. Therefore, it is important to keep the registration error as low as possible. The registration error in our study is lower than in previous publications that used a similar approach (3.0 ± 1.9 mm [15] and 4.6 ± 3.6 mm [12]), although there is no generally accepted cutoff we consider the registration error to be well within the acceptable range.

The overall model based on multiple EMM parameters shows a good predictive ability to identify areas with scar on LGE-MRI. From Fig. 2, we can appreciate that the predictive accuracy was very good in most animals. In one subject (animal 5), the model performed moderately. This might be due to a residual registration error. In one of the animals (subject number 1), all UV values (both in healthy and infarcted myocardia) were significantly lower than the values of the rest of the dataset while BV, LLS, and LAT values were within normal ranges. The reason for this is unknown. Accurate prediction in this animal was preserved because UV values were still consistently lower within scar than within healthy tissue. Previous research demonstrated poor overlap between the individual EMM-derived parameters and LGE-MRI derived TM. However, due to the dichotomization of the TM and use of a logistic mixed model used in this study, direct comparison between the two studies is not possible.

In this study, we used LGE-MRI as the gold standard for scar detection. Therefore, we could not perform a comparison between accuracy in detecting scar between LGE-CMR and our model. Despite that the NOGA-derived measurements of local mechanical activity and MR-derived values of local mechanical activity were statistically correlated, they showed at best very weak correlations. Even though circumferential strain and LLS are fundamentally different quantities, both are thought to represent local mechanical myocardial [29] function. In previous research, both were shown to correlate with the myocardial scar on LGE-MRI [16, 30]. It is therefore surprising to find only a weak correlation between these parameters. Although we can never exclude registration error as a possible explanation, the performance of our scar prediction model makes that unlikely to be the only explanation. Another possibility is that, MRI-FT, even with its reasonable agreement with MRI tagging [31], has difficulties tracking features in the circumferential direction since in the circumferential direction there is less contrast in the direction of the myocardial deformation. A more in-depth evaluation of the relationship between strain and LLS is an interesting topic for future research.

In human subjects, the normal value for the global circumferential strain is estimated to be − 23% (95% CI − 24.3 to − 21.7%) [32]. Reference ranges for endocardial circumferential strain in pigs (either in healthy pigs or in a porcine model of MI) have not been previously described. In the literature, various thresholds for circumferential strain have been reported to differentiate between infarcted and non-infarcted segments, for example, Ogawa et al. found a sensitivity of 72% and a specificity of 71% at a cutoff of – 11.2% [16, 33]. Our group has previously shown FT-derived strain has moderate ability to discriminate healthy from scarred myocardium [34]. In the current study, absolute values for endocardial circumferential strain were higher than expected within both healthy and infarcted myocardia. However, the scar area presented lower average circumferential strain values than in the healthy myocardial segments. Analysis of the diagnostic capability of endocardial circumferential strain to detect MI was not performed.

We calculated circumferential strain in 48 segments per short-axis slice, compared with the usual practice of defining the 6 anatomical cardiac segments per short-axis slice. This method allows us to compare strain parameters with increased accuracy, but is also more sensitive to errors in the FT analysis, segmentation, and image registration. Furthermore, recent studies have shown that intervendor agreement and intravendor reproducibility for MRI strain analysis are at most reasonable [35, 36].Comparing strain results from this study with results from studies in literature and previous studies from our department may, therefore, not be comparable and must be performed with caution.

### Limitations

The animal model used in this study provides large transmural myocardial infarcted areas resulting in small areas with non-transmurally infarcted tissue. Therefore, we excluded the non-transmurally infarcted tissue and dichotomized the resulting measurements to fit a multivariable mixed-effects model. This model predicts the likelihood of finding an infarction at a given location based on all EMM parameters. In Fig. 5, we show the relation between the predicted transmurality and presence of scar as a continuous variable. It seems plausible that intermediate likelihood for the presence of scar predicted by our model correspond to non-transmural infarction on MRI, but the model was not calibrated toward those predictions and we were not able to verify the performance of the model in areas with intermediate scar transmurality.

Future research should evaluate the performance of our model in a clinical dataset. In a clinical dataset, a linear model and a dataset which includes non-transmural infarctions might be better suited to predict areas with non-transmural infarction.

### Clinical Implications

LGE-MRI is considered the gold-standard imaging tool for localization of myocardial scar. However, MRI is unavailable in many cardiac disease patients, such as in patients with an implantable cardioverter defibrillator or in patients with advanced renal failure. Furthermore, when pre-procedural LGE-MRI images are available, tools for intraprocedural image fusion are not widely available. Modern EAM systems provide the functionality to derive the cardiac anatomy from pre-procedural imaging (e.g., cardiac MRI) but scar information is not extracted. Therefore, electrophysiologists often use EAM to identify scarred regions of the myocardium based on bipolar voltage. A more elaborate model, using all parameters gathered during the mapping procedure, may improve scar identification. The presented scar prediction model enables more accurate differentiation between healthy and infarcted myocardia based on a combination of all EMM parameters and may be instrumental in improving cardiac procedures such as application of regenerative therapy, ablation of ventricular arrhythmia, or cardiac biopsy. For example, several studies have proposed the use of EAM to guide endocardial biopsy procedures and showed improved biopsy yield compared with the X-ray guided approach [37, 38]. Both studies highlighted the differences in identification capabilities of diseased myocardium by different EAM parameters and suggest a combination of multiple parameters to further improve identification of diseased or infarcted myocardium. Furthermore, EMM is the clinical standard for trans-endocardial delivery of cardiac regenerative therapy into the myocardial infarct border zone [39] and continues to be used in multiple current studies [3]. A recent publication suggested that EAM-guided LV lead implantation improves response to cardiac resynchronization [40]. An additional advantage is the ability to directly identify the area of latest activation on the LV endocardium using EAM during the implantation.

## Conclusion

The scar prediction model, based on the combination of unipolar voltage, bipolar voltage, local activation time, and linear local shortening by NOGA, can accurately distinguish areas with MI from healthy myocardium as defined by LGE-MRI. In this dataset, unipolar voltage and bipolar voltage were the strongest predictors for the presence of MI. In the future, the scar prediction model may prove to be useful in VT ablations, biopsy procedures, or regenerative therapy. Surprisingly, EMM-derived parameters were not significantly correlated with MRI-derived strain parameters.

## Electronic Supplementary Material

ESM 1 (DOCX 20 kb)

## Abbreviations

BV: bipolar voltage
EAM: electroanatomical mapping
EMM: electromechanical mapping
FT: feature tracking
FWHM: full-width at half maximum
ICP: iterative closest point
LAT: local activation time
LGE: late gadolinium enhancement
LGE-MRI: late gadolinium–enhanced magnetic resonance imaging
LLS: local linear shortening
LV: left ventricle
MI: myocardial infarction
MRI-FT: magnetic resonance imaging feature tracking
OR: odds ratio
Strain_max_: maximal endocardial circumferential peak strain
*T*_onset_: timing of onset of shortening
TM: infarct transmurality
TTP_max_: time to maximal endocardial circumferential peak strain
UV: unipolar voltage
WT: wall thickening
WT%: fractional wall thickening
