# Supplementary material for: 3D Myocardial Scar Prediction Model Derived from Multimodality Analysis of Electromechanical Mapping and Magnetic Resonance Imaging
Source: J Cardiovasc Transl Res. 2019 Jul 23;12(6):517–27. doi: 10.1007/s12265-019-09899-w (PMC6854049; doi:10.1007/s12265-019-09899-w)
Supplement: Supplementary file 1 — (DOCX 20 kb) [file 12265_2019_9899_MOESM1_ESM.docx]

# Supplementary information 1

## MRI parameters

In eleven animals MRI acquisition was performed using a clinical 1.5T scanner (Ingenia TX, Philips Healthcare, Best, the Netherlands). BTFE acquisition parameters: Repetition time [TR]/echo time [ET] = 3.39 ms/1.69 ms. Flip angle = 60°, Pixel size = 1.25 x 1.25 mm, Field of view [FOV] = 320 x 320 mm, 256 x 256 matrix, 8 mm slice thickness, 30 phases/R-to-R interval. LGE acquisition parameters: [TR]/[ET] = 3.64 ms/1.19 ms. Flip angle = 25°, Pixel size = 1.25 x 1.25 mm, [FOV] = 296 x 296 mm, 256 x 256 matrix, 4 mm slice thickness.

In four animals MRI acquisition was performed using a clinical 3T scanner (Achieva TX, Philips Healthcare, Best, the Netherlands)[1]. The BTFE acquisition parameters were as follows: Repetition time [TR]/echo time [ET] = 3.2 ms/1.6 ms. Flip angle = 45°, Pixel size = 2 x 2 mm, Field of view [FOV] = 320 x 320 mm, 256 x 256 matrix, 8 mm slice thickness, 30 phases/R-to-R interval.

### Timing of onset algorithm

The algorithm automatically determines the timing of onset shortening, defined as the beginning of the downslope of the endocardial circumferential strain curve. In this study, a modified version of the algorithm for estimation of onset time of shortening described by Zwanenburg *et al* was used[2]*.* In this study instead of MR tagging, the curves were calculated using MR feature tracking on standard cardiac cine images triggered on the R-to-R interval. Thus omitting the necessity to correct for a strain offset caused by variations in tag distance nor to account for the dependency of the accuracy on the tagging contrast. The line fit model *L* was defined as described by Zwanenburg *et al*. Akinetic segments with a circumferential strain amplitude of >-7.5% were excluded from the analysis. The strain curves were interpolated using the *spline* function. The endpoint of the time interval was defined as the moment of peak strain rate. The line fit model, *L*, was fitted to the automatically selected data with a step size of 1 ms. The onset time of shortening T_onset_ was defined by the timing corresponding to the lowest residual error (sum of squares).

# Supplementary information 2

## Performance of the prediction model

Performance of a model can be assessed using measures for discrimination and calibration. Discrimination gives an indication of how well the model can classify patients in those with and those without the outcome. The most commonly used measure for discrimination is the c-statistic, which for a binary problem is equivalent to the area under the receiver operating curve. A c-statistic of 0.5 indicates that the model performs as well as a coin flip, while a value of almost 1 indicates perfect discrimination. Calibration, on the other hand, gives an indication of the agreement between predicted and observed outcomes. This means that if the model predicts a 50% chance of the outcome, that for a sample of 100 data points the observed frequency of the outcome is 50. Calibration can be assessed with a calibration slope, which should be 1 for a perfect calibration. A calibration slope larger than 1 in the validation step is an indication of overfitting[3, 4].

For a mixed-effects model, the overall performance measures are not appropriate, as they do not take the clustering of the data into account. Therefore, the c-statistic is derived within each cluster or subject and then the average, weighted for the amount of data point within each cluster, is used[5]. The within-subject calibration slope is assessed by fitting a new mixed-effects logistic regression model, with as only input the linear predictor from the original model and a random intercept and slope[6].

## Internal validation

The performance measures described above can be overly optimistic when determined in the sample where the model is based on. We can correct for this optimism by refitting the model repeatedly in new bootstrap samples and subsequently validate it in the original sample. The difference between c-statistic and calibration slope in the bootstrap and the original sample is called the optimism and will be subtracted from the values derived in the development set. In this paper, 500 bootstrap samples are used for this step.

For a mixed-effects model, bootstrap sampling can be done by sampling individual data points, whole clusters of data or a 2-step approach where both sampling methods are combined. If there are less than 20 clusters present, the regular bootstrap scheme with individual data points is most appropriate[7]. External validation in a new population remains necessary to verify the performance of the model in related populations.

# References supplemental data

1. van Es R, van Slochteren FJ, Jansen of Lorkeers SJ, et al (2016) Real-time correction of respiratory-induced cardiac motion during electroanatomical mapping procedures. Med Biol Eng Comput 54:1741–1749 . doi: 10.1007/s11517-016-1455-3
2. Zwanenburg JJM (2004) Timing of cardiac contraction in humans mapped by high-temporal-resolution MRI tagging: early onset and late peak of shortening in lateral wall. AJP Hear Circ Physiol 286:H1872–H1880 . doi: 10.1152/ajpheart.01047.2003
3. Harrell FE, Lee KL, Mark DB. Tutorial in biostatistics multivariable prognostic models: Issues in developing models, evaluating assumptions and adequacy, and measuring and reducing errors. Stat Med [Internet]. 1996 Feb 29 [cited 2018 Sep 24];15(4):361–87. Available from: http://www.ncbi.nlm.nih.gov/pubmed/8668867
4. Steyerberg EW, Vickers AJ, Cook NR, Gerds T, Obuchowski N, Pencina MJ, et al. Assessing the performance of prediction models : A framework for some traditional and novel measures. Epidemiology. 2010;21(1):128–38.
5. Oirbeek R Van, Lesaffre E. Assessing the predictive ability of a multilevel binary regression model. Comput Stat Data Anal [Internet]. 2012;56(6):1966–80. Available from: https://doi.org/10.1016/j.csda.2011.11.023
6. Van Klaveren D, Steyerberg EW, Perel P, Vergouwe Y. Assessing discriminative ability of risk models in clustered data. BMC Med Res Methodol. 2014;14(1):1–10.
7. Bouwmeester W, Moons KGM, Kappen TH, van Klei WA, Twisk JWR, Eijkemans MJC, et al. Internal validation of risk models in clustered data: a comparison of bootstrap schemes. Am J Epidemiol [Internet]. 2013 Jun 1;177(11):1209–17. Available from: http://www.ncbi.nlm.nih.gov/pubmed/23660796

Supplemental **Table 1**. Results from the relations between MRI-derived wall thickening, timing and strain analysis parameters and electromechanical parameters: local activation time and linear local shortening.

| Determinant | Outcome | Coefficient | Lower CI | Upper CI | P | R^2^ |
| --- | --- | --- | --- | --- | --- | --- |
| T_onset_ | LAT | 0.02 | 0.004 | 0.05 | 0.05 | 0.006 |
| TTP_max_ |  | 0.01 | 0.004 | 0.02 | 0.02 | 0.014 |
| WT | LLS | 0.80 | 0.46 | 1.13 | < 0.01 | 0.029 |
| WT% |  | 0.05 | 0.03 | 0.08 | < 0.01 | 0.026 |
| strain_max_ |  | -0.15 | -0.21 | -0.09 | < 0.01 | 0.023 |

CI: confidence interval; LAT: local activation time; LLS: linear local shortening; strainmax: peak strain; Tonset: timing of onset of shortening; TTPmax: time to peak shortening; WT: wall thickening; WT%: fractional wall thickening.
